# Is aberrant CD8+ T cell activation by hypertension associated with cardiac injury in severe cases of COVID-19?

**DOI:** 10.1038/s41423-020-0454-3

**Published:** 2020-05-12

**Authors:** Chao Zhang, Fu-Sheng Wang, Jean-Sébastien Silvestre, Fernando Arenzana-Seisdedos, Hong Tang

**Affiliations:** 1 Treatment and Research Center for Infectious Diseases, The Fifth Medical Center of PLA General Hospital, National Clinical Research Center for Infectious Diseases, Beijing, China; 2 Inserm UMRS 970, Paris Centre de Recherche Cardiovasculaire (PARCC), 75737 Paris cedex 15, Cedex, France; 3 CAS Key Laboratory of Molecular Virology and Immunology, COVID-19 Response Team, Institut Pasteur of Shanghai, Chinese Academy of Sciences, Shanghai, China

**Keywords:** Inflammation, Viral infection

COVID-19 global pandemic, caused by severe acute respiratory syndrome coronavirus type 2 (SARS-CoV-2),^1^ has swept 185 countries and regions with more than 2,824,728 confirmed cases, and 197,667 death as on April 25, 2020 according the Coronavirus Resource Center at Johns Hopkins University. Accumulating data suggest that hypertension, diabetes, and cardiovascular diseases are the most frequent comorbidities in COVID-19 patients, and case mortality rates tended to be high in these individuals.^2^ Among few studies that focus on COVID-19 severe pneumonia, cardiovascular diseases are among the most frequent comorbidities,^3–5^ with hypertension being the most common (58 of 191 patients, 30%) in one study, exceeding twofold in COVID-19 ARDS patients (23 of 84, 27.4%) more than mild patients (16 of 117, 13.7%) in another study.

Angiotensin II (Ang II) is a potent hypertensive hormone, and increased Ang II is associated with hypertension and heart failure,^6^ lung^7^ and renal dysfunction.^8^ Angiotensin-converting enzyme 2 (ACE2) converts Ang II to Ang 1–7 to negatively regulate the renin–angiotensin system (RAS) and renin–angiotensin–aldosterone system.^9^ SARS-CoV-2 binds to the catalytic domain of ACE2, with higher binding affinity than SARS-CoV, for cell entry.^10–12^ Notably, SARS-CoV Spike protein engagement can downregulate ACE2 expression and activate RAS for lung injury.^13^ Furthermore, plasma level of Ang II is markedly elevated and correlated to viral load and lung injury of COVID-19 patients.^14^ Therefore, reduction of cell surface ACE2, due to SARS-CoV-2 endocytosis, would augment Ang II pathological processes in the development of hypertension, cardiomyopathy, and nephropathy^15^ in severe COVID-19 patients.

Hypertension is treated with ACE inhibitors and angiotensin II type-I receptor blockers (ARBs), resulting in ACE2 upregulation. It is unclear whether ARB/ACE regime is warranted in COVID-19, due to insufficient evidence at the moment. European Society of Cardiology recommends not to change RAS blockade in COVID-19 patients who are on it, unless adverse clinical indications occur. Further study needs to better understand the impaired RAS in the viral pathogenesis of COVID-19.

ACE2 is highly expressed in the heart tissue, implicating a possibly direct viral infection of the myocardium. Strikingly, two independent postmortem examinations revealed no evidence of viral infection or replication in cardiac tissues, albeit pronounced cardiac inflammation exists.^16,17^ It is unlikely that viral infection and replication directly cause or aggravate cardiac injury in these severe patients. It is becoming recognized that macrophages and T cells infiltrate to the heart in response to hypertension, and the end-organ damage are in part mediated by activation of these infiltrated cells.^18^ Our lab showed that mice lacking CD8+ T cells are efficiently protected from hypertension-induced cardiac damage. CD8+ T cells thus can sense the hypertension independent of T cell receptor.^19^ More importantly, CD8+ T cells are required for macrophage infiltration in myocardium and subsequent activation by CD8+ T cells secreted IFN-γ.

How do CD8+ T cells respond to hypertension? One study suggests that mineralocorticoid receptor on CD8+ T cells directly sense blood pressure and promote inflammatory milieu through secreting IFN-γ.^20,21^ Furthermore, hypertension can trigger oxidative modification of proteins in DC cells by highly reactive γ-ketoaldehydes (isoketals), which activate DC to produce IL-6, IL-1β, and IL-23. Activated DCs promote T cell, particularly CD8+ T cell, proliferation and production of IFN-γ and IL-17A.^22^ Intriguingly, a secondary hemophagocytic lymphohistiocytosis, which associates with a massive CD8+ T cell and macrophage activation but decreased NK cell activity, has been noted for COVID-19 patients in European ICUs. Taken together, these results suggest that CD8+ T cells may function as a key hypertension effector that drives macrophage-mediated cardiac damage.

Severe COVID-19 patients also showed increased IL-6, IL-1β, and IFN-γ.^23^ It is worthy of studying whether blockade of IL-6 or IL-1β, which is currently under clinical trials, would reduce cardiac injury through inhibition of CD8^+^ T cell-macrophage infiltration and overactivation. The glucocorticoid treatment of ICU patients shall also be closely monitored for potential beneficial or detrimental effect on CD8^+^ T cell activation. Lastly, CCR5 is a major chemoattracting receptor in CD8^+^ T cells that involves in various pathogenic conditions, including viral infections.^24^ The antiviral drugs, such as Selzentry (maraviroc) and Leronlimab (PRO 140), have been successfully used for treatment of AIDS.^25^ It is therefore of great interest to study whether these drugs can block cardiac infiltration of CD8^+^ T cells thereby reduce hypertensive cardiac injury of COVID-19 patients.
